# Modeling Fragile X Syndrome Using Human Pluripotent Stem Cells

**DOI:** 10.3390/genes7100077

**Published:** 2016-09-28

**Authors:** Hagar Mor-Shaked, Rachel Eiges

**Affiliations:** Stem Cell Research Laboratory, Medical Genetics Institute, Shaare Zedek Medical Center Affiliated with the Hebrew University School of Medicine, Jerusalem 91031, Israel; hagar.mor@mail.huji.ac.il

**Keywords:** fragile X syndrome, *FMR1* gene, FMRP, human embryonic stem cells, disease modeling, patient-derived iPS cells, epigenetics, repeat somatic instability, neurodevelopment

## Abstract

Fragile X syndrome (FXS) is the most common heritable form of cognitive impairment. It results from a loss-of-function mutation by a CGG repeat expansion at the 5′ untranslated region of the X-linked *fragile X mental retardation 1 (FMR1)* gene. Expansion of the CGG repeats beyond 200 copies results in protein deficiency by leading to aberrant methylation of the *FMR1* promoter and the switch from active to repressive histone modifications. Additionally, the CGGs become increasingly unstable, resulting in high degree of variation in expansion size between and within tissues of affected individuals. It is still unclear how the FMR1 protein (FMRP) deficiency leads to disease pathology in neurons. Nor do we know the mechanisms by which the CGG expansion results in aberrant DNA methylation, or becomes unstable in somatic cells of patients, at least in part due to the lack of appropriate animal or cellular models. This review summarizes the current contribution of pluripotent stem cells, mutant human embryonic stem cells, and patient-derived induced pluripotent stem cells to disease modeling of FXS for basic and applied research, including the development of new therapeutic approaches.

## 1. Introduction

Fragile X syndrome (FXS; OMIM #300624) is the most common heritable form of cognitive impairment (1 in 4000 male and 1 in 8000 female births), and is the leading known genetic cause of autism. It is inherited as an X-linked trait and results from a deficiency in the fragile X mental retardation protein (FMRP) [1]. FMRP is an RNA-binding protein that is important for the transport, stabilization and translation of messenger RNA (mRNA) into proteins that affect synaptic plasticity and connectivity in the central nervous system. In the absence of FMRP the dendritic spines are longer, thinner and less mature [2].

Nearly all FXS patients lack FMRP due to an unusual loss-of-function mutation; namely, a CGG tri-nucleotide microsatellite repeat expansion in the 5′ untranslated region (5′-UTR) of the X-linked *fragile X mental retardation 1 (FMR1)* gene [3,4,5]. While in most people the number of CGGs is stable and ranges from 5 to 55 units, in patients it abnormally expands to over 200 units (full mutation; FM) [6]. When the CGG repeats range from 55 to 200 (permutation; PM) they tend to further expand in the female germ line (germ line instability), resulting in the birth of affected children. Once the CGGs reach the FM range, they lead to the spread of abnormal 5′-C-phosphate-G-3′ (CpG) methylation and repressive histone modifications at the 5′ regulatory region, resulting in epigenetic gene silencing of *FMR1* [3,6,7,8,9,10,11,12]. In addition, the CGGs become increasingly unstable, leading to a high degree of variation in expansion size between and within tissues of affected individuals [3,13]. It is still unclear how the CGG expansion leads to epigenetic gene silencing of the *FMR1* in FXS patients, and how the loss-of-function of FMRP leads to neural deficits in FXS fetuses. In addition, the underlying mechanisms for germ line and somatic CGG instabilities in FXS are unknown. In terms of treatment, it would be enormously beneficial to find how/whether *FMR1* gene inactivation can be reversed in patients. As is the case for other pathologies resulting from untranslated repeat expansions, modeling FXS can be particularly challenging since it is difficult to clone lengthy repetitive elements. Nevertheless, numerous transgenic and knockin animal models of FXS have been created [14,15,16,17,18,19,20,21,22]. Unfortunately, none of the currently available animal models accurately or completely reproduce the molecular and cellular phenotypes that are typically observed in patients [23,24,25]. These crucial issues underscore the importance of developing alternative biological systems, preferably human-based, that can reproducibly recapitulate the different facets of the disease. 

Human embryonic stem cells (hESCs) are undifferentiated pluripotent cell lines with unrestricted self-renewal and the ability to differentiate into a wide range of cell types [26]. They are established from in vitro fertilization (IVF) embryos, and can be obtained from genetically affected embryos following preimplantation genetic diagnostic procedures [27]. The derivation of hESCs directly from diseased embryos constitutes a powerful tool for disease modeling (reviewed in [28]) by affording the exploration of disease relevant tissues and/or developmental stages that are otherwise inaccessible to research. This is particularly beneficial in FXS, where studies are often limited to aborted fetuses, adult postmortem brain samples, or disease irrelevant tissues. 

Thus far, over a dozen of FXS hESC lines have been established worldwide (see Table 1) and utilized to address some of the questions presented above. However, the availability of those exceptional cell lines is a limiting factor since it relies on the acquisition of genetically affected embryos, a valuable resource that is accessible to only a small number of stem cell deriving laboratories and requires teamwork with a preimplantation genetic diagnosis (PGD) performing center. An alternative approach to generating diseased pluripotent stem cells (PSCs) entails the de-differentiation of adult tissues by overexpressing a limited number of transcription factors [26]. Quite remarkably, the resulting cell lines, termed induced pluripotent stem cells (iPSCs), are indistinguishable from embryo-derived hESC lines by most measures; i.e., gene expression and developmental potential [29,30]. The advantages of this technique are obvious. First, it bypasses the limited pool of affected human embryos for hESC line derivation. Second, it can serve as a powerful tool for disease modeling, in that iPSCs can be easily derived from somatic cells of patients by any stem cell investigating lab. In fact, many mutant iPSC lines have already been established for FXS, and is the main approach for creating human-based disease models of dynamic mutations that otherwise cannot/are difficult to artificially induce in hESCs [31,32,33,34,35,36,37,38,39]. Nevertheless, despite the great similarity between hESCs and iPSCs, there are some discrepancies between both cell types which can be crucial and should be taken in to consideration when addressing unresolved questions related to FXS.

This review summarizes the current contribution of mutant PSCs, hESCs and patient-derived iPSCs to research of FXS. For each we summarize the currently available stem cell based models, highlight how they have contributed to better understanding of the related mechanism, their limitations and the ways in which they can be utilized in future investigations.

## 2. Exploring the Epigenetics of FXS

Despite the tight correlation between transcriptional inactivation and chromatin compaction, the precise mechanism governing the silencing of *FMR1* is not fully understood. In addition, it remains unclear when *FMR1* gene inactivation takes place during embryo development. Previous reports related to the timing of *FMR1* silencing showed that the *FMR1* locus is still active at 8–10 weeks of gestation. It begins to be repressed in fetal tissues by the end of the first trimester, and frequently remains partly active in extra embryonic tissues [46,47,48,49,50]. This led to the generalized view that *FMR1* inactivation occurs through a developmentally regulated process. These studies also provided evidence that the CGG expansion is a necessary, but not sufficient condition for gene inactivation and that additional differentiation-dependent factors are required to achieve *FMR1* silencing. When the first male FXS hESC line was established (HEFX), it was found to express *FMR1* mRNA at levels comparable to wild type (WT) hESCs and to be completely unmethylated at *FMR1* [27]. This validated the widespread assumption that *FMR1* epigenetic silencing is triggered by differentiation and encouraged investigators to posit that the cellular phenotype of FXS neurons could be corrected simply by removing the repressive epigenetic marks that are incorrectly acquired by cell de-differentiation. However, when patients’ skin fibroblasts were reprogrammed using standard reprogramming procedures, *FMR1* remained inactive and hypermethylated in iPSCs derived from FXS fibroblasts [32,33,36,38,39]. This suggests that once the incorrect epigenetic marks are established, they are stable and irreversible, or at least resistant to the current reprogramming methods. Interestingly, when De Esch and colleagues converted fibroblasts into iPSCs from an atypical individual who carries an unmethylated full expansion with an active *FMR1* gene, it recurrently resulted in hypermethylation and complete inactivation of FMR1 in the newly established iPSC clones [34]. This led to the conclusion that transcription factor reprogramming may incorrectly hypermethylate this region provided that the CGGs are sufficiently long. Furthermore, it highlighted the distinction between FXS hESCs and iPSCs in disease modeling, by drawing attention to the limitations involved in using mutant iPSCs when investigating the mechanism underlying epigenetic gene silencing of *FMR1*.

**Figure 1:**
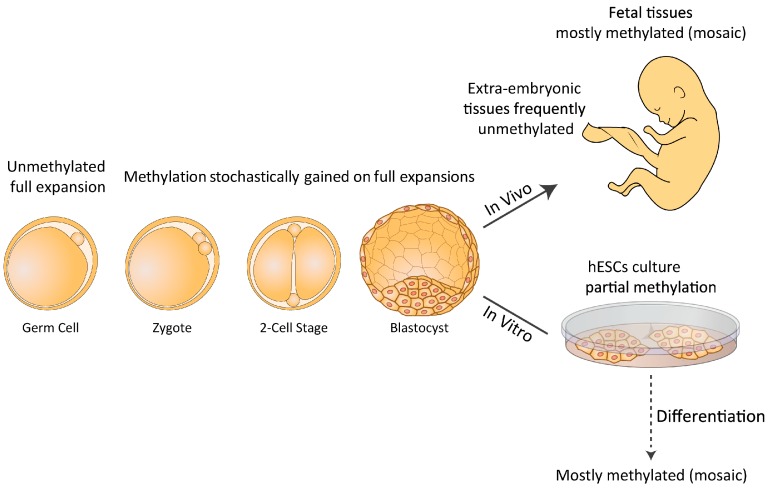
Model for timing and nature of *fragile X mental retardation 1 (FMR1)* hypermethylation in Fragile X syndrome (FXS) human embryonic stem cells (hESCs). Abnormal methylation is first achieved on full expansions in FXS at a restricted time point before/during embryo implantation. Once established, it is irreversible and is clonally maintained. CGG expansions that escape abnormal methylation during this limited time frame remain unmethylated or become further inactivated by a second wave of de novo methylation later during differentiation. Mostly methylated (mosiacism) means that some of the cells are methylated and that some are not.

As more FXS hESC lines were made available, it became apparent that *FMR1* hypermethylation is not restricted to somatic cells but can also be acquired in the undifferentiated state, since studies have shown that FXS hESC lines are fairly heterogeneous for methylation levels despite the presence of a full mutation [32,51]. In fact, of the 11 FXS hESC so far examined, the majority (7) have presented certain levels of *FMR1* hypermethylation (see Table 1), ranging from 0% to 65% upstream to the repeats as measured by bisulfite pyro-sequencing [32]. Methylation levels remain unchanged over time in culture (more than 10 successive passages) and are tightly coupled with a change in histone modifications, a loss in H3K4me2 (active mark) and a gain in H3K9me3 (repressive mark). Studies at the resolution of individual DNA molecules showed that the methylation states of full mutations are binary; either they are completely methylated or they are entirely unmethylated [32]. As *FMR1* methylation levels remain unchanged over time in culture in those particular cell lines, unmethylated full expansions most likely arise from imperfect de novo methylation rather than from a failure to reproducibly maintain aberrant methylation patterns. This may suggest that the expansion is not consistently methylated in all the cells of the pre-implantation embryo. Alternatively, *FMR1* inactivation may initially be set by the beginning of gastrulation; i.e., in the post-implantation epiblast stage. In the last few years it has been argued that hESCs, unlike mouse embryonic stem cells (ESCs), more closely resemble epiblast cells (primed ESCs) than inner cell mass (ICM) cells (naïve ESCs) [52,53,54]. If so, they are predicted to reflect a less primitive ground state of pluripotency and exhibit higher methylation levels than originally assumed. By growing these cells in conditions that favor naïve over primed hESC cultures, Gafni et al. claimed they were able to re-activate the *FMR1* gene in FXS iPSCs [52]. However, under the same growth conditions, other researchers have been unable to reverse/prevent hypermethylation of the *FMR1* in FXS hESC/iPSCs clones, respectively [34]. Hence it is essential to explore whether FXS pre-implantation embryos are already *FMR1* methylated, and if so whether *FMR1* silencing can be achieved before the blastocyst stage. If methylation is found, it would be valuable to assess the extent of its variation within affected embryos. In any case and regardless of the exact time at which hypermethylation is first established, the failure to methylate expansions greater than 200 CGGs may point to a “window of opportunity” within which aberrant methylation can occur such that expansions that have coincidentally escaped de novo methylation persist. A model that relates to the timing and nature of *FMR1* hypermethylation suggests that abnormal methylation is first acquired on full expansions in FXS at a restricted time point before/during embryo implantation. Once established, it is irreversible and is clonally maintained. Expansions that escape abnormal methylation during this limited time frame remain unmethylated or become inactivated later during differentiation (see Figure 1). The relatively high rate of methylation mosaicism among affected individuals is consistent with this model, and is in line with the majority expressing significant levels of *FMR1* mRNA [55].

Mechanistically, researchers are attempting to address the question of how CGG expansion leads to de novo methylation of the 5′-UTR of *FMR1* by taking advantage of FXS hESCs which, unlike the somatic cells of patients and patient-derived iPSCs, provide an unusual opportunity to uncouple CGG expansion and heterochromatin induction in a developmentally regulated context. Given the accumulated data related to epigenetic gene silencing in other loci, particularly at repetitive elements, there is a common view that *FMR1* inactivation is elicited in a way that is RNA-directed [56,57,58]. Over the last few years several models have been proposed to explain how *FMR1* silencing is achieved, although none have yet been validated. One model put forward by Ladd et al. argued that the insulator-binding protein CTCF differentially binds to the flanking regions of the repeats in normal but not expanded alleles [59,60]. Furthermore, an overlapping gene that transcribes in an antisense orientation and extends across the CGG repeats was identified (*ASFMR1*) [59,60]. Consistent with these findings and established data related to CTCF regulated double-strand RNA (dsRNA)-mediated gene silencing in other loci [61,62,63,64], it was hypothesized that when the CGGs expand beyond 200 repeats, this leads to the loss of CTCF binding adjacent to the repeats, and as a results to inappropriate heterochromatin induction and transcriptional silencing of *FMR1*. To find the potential mechanistic link between *FMR1* inactivation and CTCF binding, Avitzour et al. took advantage of FXS hESCs and examined whether methylation is coupled with the binding loss of CTCF adjacent to the repeats [32], as suggested earlier [59,60]. However, no enrichments for CTCF could be detected by chromatin immunoprecipitation (ChIP) analysis in WT or affected *FMR1* unmethylated hESCs. This excluded the possibility of CTCF binding loss as a trigger for heterochromatin by CGG expansion. However it does not rule out the potential role of bidirectional transcription as a mediator of silencing, given the capacity of long dsRNA to be processed into small RNA molecules by the RNA interference (RNAi) machinery [65]. Another proposal is that the trigger for silencing is not mediated by antisense transcription but rather depends on the propensity of CGG-expanded RNAs to fold into hairpin structures, thus providing a preferential substrate for *Dicer* activity [66,67]. Regardless of the source, it is hypothesized that by pairing either to nascent RNA transcripts or directly to the DNA sequence, the Dicer processed short repeat containing RNAs attract silencing complexes (like DNA de novo methylatransferase and histone methyltransferases) to the complementary genomic region, and in doing so lead to the induction and spread of heterochromatin (similar to that described for pericentromeric regions [68,69]). By contrast, knockdowns of *Dicer*, *Ago1* or *Ago2* did not prevent *FMR1* epigenetic gene silencing in FXS hESCs [51], which implies that FMR1 gene inactivation may not depend on the RNAi pathway as previously suggested [31].

Other studies exploring the mechanism of *FMR1* gene silencing involving FXS hESCs have pointed to the role of cell differentiation in this process. For instance, Colak and colleagues, who employed two FXS hESC lines (WCMC-37 and SI-214) reported they had uncovered an mRNA-mediated mechanism that drives epigenetic gene silencing in a way that relies on neuronal differentiation [51]. They claimed that the lengthy CGG *FMR1* mRNA hybridize to the complementary CGG-repeat portion of the *FMR1* gene to form an RNA:DNA duplex. They demonstrated that upon directed differentiation into nerve cells, the destruction of the naturally formed RNA:DNA hybrids at the 5′-UTR of the *FMR1* locus is consistent with the repression of *FMR1* transcription, and is coupled with a switch from active to repressive histone marks (H3K4me2 and H3K9me2, respectively). This could suggest that RNA:DNA hybrid formation at a critical time point during cell differentiation prevents neural-induced *FMR1* silencing. However, a careful examination of the methylation status of *FMR1* in both FXS hESC lines indicated that they were partly methylated to begin with. It remains to be determined whether *FMR1* inactivation by neuronal differentiation reflects a second wave of gene inactivation. In addition, it is unclear whether silencing in FXS undifferentiated cells is achieved by the same mechanism as in differentiating cells, and whether the block in *FMR1* transcription in FXS HESC derived neurons coincides with DNA hypermethylation. It would be of great interest to explore the potential role of R-loops (three-stranded nucleic acid structures formed between an RNA strand to one of the DNA strands) in maintaining the *FMR1* in a transcriptionally active configuration, in light of recent findings on their potential role as a mechanism protecting against epigenetic gene silencing [70]. Conversely, they may attract silencing complexes when they become abnormally stabilized, as was recently suggested for Friedreich’s ataxia at the *FXN* gene [71].

A different feature related to epigenetic *FMR1* inactivation raises the issue of whether the inactive state is maintained once it is achieved. For years, the question was whether the maintenance of the inactive state depends on the presence of the CGG expansion, or whether once established it is endlessly preserved (similar to the effect of *X-inactive specific transcript* (*XIST*), which is essential for initiation and spread but is not needed for the maintenance of X inactivation) [72]. However, the low efficiency of targeted mutagenesis in somatic cells, together with considerable difficulty of targeting particularly long repetitive regions impeded researchers from running these experiments until the advent of gene editing. Taking advantage of hESCs/iPSCs as a powerful tool for gene manipulation, Park and colleagues aimed to remove the CGG repeats from the *FMR1* gene in male FXS iPSCs [73]. Using the Clustered regularly interspaced short palindromic repeats/CRISPR associated protein 9 (CRISPR/Cas9) approach they managed to restore *FMR1* gene expression and FMRP protein levels in a pair of clones by targeting a deletion at the CGG repeats and immediate 5′-flanking region that led to nearly complete reversion of hypermethylation (≤10%) concomitant to the gain of H3K4me3 and the partial loss of H3K9me2 marks. Demethylation and re-activation of the *FMR1* led to sustained expression through long-term differentiation into mature neurons. These findings suggest that the mechanism responsible for maintaining the inactive state does not depend on epigenetic memory, but rather on persistent de novo acquisition of abnormal epigenetic marks conditioned by the presence of the expansion at each cell division. If indeed the expanded repeats are crucial for the maintenance of aberrant DNA hypermethylation at each cell cycle, it would be stimulating to investigate which molecular mechanism enables this process and how is it mediated.

## 3. CGG Repeat Instability

Repeat instability describes a mutational event that refers to the addition or deletion of DNA repeats arranged in tandem. It accounts for over 20 neuromuscular, neurodevelopmental and neurodegeneration pathologies, including FXS (for comprehensive review see [74]). In *FMR1*, when the CGGs increase beyond the normal range (>55 CGGs) they tend to further expand when transmitted through the female germ line (germ line instability). Once the CGGs expand over 200 repeats, they lead to FXS and become increasingly unstable. This results in a high degree of variation in expansion size between and within tissues of affected individuals, a phenomenon termed repeat somatic instability [75,76]. In FXS, this event is considered to be developmentally regulated, since it is largely restricted to the early stages of embryo development [77,78]. While in FXS the clinical importance of this mutational event is uncertain, it contributes to the severity of the disease in other unstable repeat disorders [79,80]. Therefore, understanding the mechanism of repeat instability in FXS may shed light on the pathogenesis of other repeat associated conditions.

The mechanism for instability, both germ line and somatic, is unknown. Evidence for the involvement of DNA replication, DNA repair and recombination has been obtained using different model systems (for a comprehensive review see [81]). Regardless of the mechanism, all the proposed instability models are based on the formation of unusual structures in the DNA that are finally resolved by the addition (expansions) or deletion (contractions) of CGG repeats. Based on the characterization of patients’ somatic cells, a correlation was reported between hypomethylation and CGG instability. Although cell fusion experiments have led to contradictory results [78,82] as to the existence of this correlation, it is becoming clear that hypermethylation most likely counteracts CGG instability in FXS cells. For example, a study that characterized a large set of FXS hESC lines by methylation-sensitive Southern blot provided evidence for a strong inverse correlation between hypomethylation and variability in expansion size [32]. Moreover, when FXS cells with hypomethylated vs. hypermethylated expansions are induced to reprogram, they give rise to isogenic FXS iPSC clones that act differently with respect to CGG stability. When the CGGs are hypermethylated, the iPSC clones present a fixed number of CGGs. On the other hand, when the CGGs are unmethylated, the iPSC clones present a wide range of expansion sizes [32,34,39]. Because iPSCs are single cell in origin, these findings provide firm evidence for the ongoing repeat instability in those cell types when the *FMR1* is hypomethylated. This supports, but still does not prove, that somatic instability may be inhibited by the gain of aberrant methylation. It would be interesting to explore whether methylation and repeat somatic instability are mechanistically associated, or whether they are two unrelated molecular events that have regulators in common. 

Evidence emerging from diverse systems, including bacterial plasmids and various replication defective strains of yeast, support the hypothesis that somatic instability in FXS is promoted by DNA replication [83,84,85,86]. Based on these experiments, a replication-based model was suggested. This model relies on the slippage of Okazaki fragments during lagging strand synthesis, and on the marked difference between complementary DNA strands in G-content, particularly in the event of a full mutation. This is due to the preferential potential of G-rich DNA strands to form stable secondary structures [87,88,89,90,91]. According to this model, if the CGGs are replicated by lagging strand synthesis, this favors the addition of repeats. On the other hand, if the CGGs are replicated by leading strand synthesis, this preferentially leads to the loss of repeats. In other words, replication direction through the CGGs dictates whether expansions (by lagging strand synthesis when origin of replication (ORI) is 3′ to the repeats) or contractions (by leading strand synthesis when ORI is 5′ to the repeats) will be induced [88]. To explain the broad heterogeneity in expansion size that is typically observed in patients and is apparently restricted to early developmental stages, it was postulated that during early embryogenesis there is a switch in ORI usage. A shift in replication direction from a downstream to an upstream ORI relative to the repeats was proposed (origin-shift model [92]). Using a pair of FXS hESC lines, Gerhardt and colleagues reported a difference in ORI usage exclusive to undifferentiated FXS cells. By applying a state-of-the-art technique for mapping replication origins and measuring fork progression at single-molecule resolution (single-molecule analysis of replicated DNA; SMARD), they proposed a switch in the replication direction across the CGGs [45]. According to their findings and unlike in WT hESCs, replication proceeds mainly from a downstream ORI in FXS hESCs, representing early embryonic cells. This indicates that replication across the CGGs is attributed entirely to lagging strand synthesis and is predicted to enhance expansions. In addition, they posited that once the cells begin to differentiate, replication patterns change and become indistinguishable from normal hESCs, suggesting that this irregularity is a developmentally regulated event that is restored with differentiation by the re-activation of an upstream ORI. Unexpectedly, Gerhardt et al. found no difference in the rate of replication fork progression at the repeats between WT and FXS hESCs, implying that repeat length or ORI position have no effect on fork stalling, as was previously shown [93]. Collectively, their findings support the origin-shift model. However, considering the presence of PM in those cell lines and the fact these cell lines are at least partly methylated, interpretation calls for caution. Furthermore, assuming the existence of a change in ORI usage with differentiation, it remains unclear whether this is a cause or an effect of the expansion, or whether it is mechanistically related to somatic instability, and if so, by what mechanism. On the other hand, evidence accumulating from mouse models and patients’ cells links repair proteins to CGG instability [94,95]. Their possible contribution should be further explored using PSCs. 

Finally, while all of the aforementioned studies deal with the use of PSCs to address fundamental issues concerning somatic instability in FXS, it would be worthwhile investigating the mechanisms underlying germ line instability; i.e., to determine how a PM converts into a FM when transmitted through the mother’s germ line. This can be accomplished once efficient protocols for inducing differentiation of hESC/iPSC into functional gametes are developed. In fact, ESCs have already been differentiated into fully matured oocytes and viable offspring produced from in vitro derived gametes in mice [96], thus paving the way for these types of experiments in humans in the near future.

## 4. Neurological Pathology in FXS Neurons Derived from hPSC

FMRP is ubiquitously expressed in the body, but is most abundant in the brain and testes. It interacts with approximately 4% of all mRNA species in the brain and is responsible for transporting them out of the cell nucleus to the synapses of neurons [97]. Most of these mRNA targets have been found to be located in the dendrites of neurons, thus providing a good explanation as to why abnormal dendritic spines are typically observed in FXS patients. FMRP has been implicated in several signaling pathways including the metabotropic glutamate receptor (mGluR) [98,99], dopamine and the gamma-Aminobutyric acid (GABA) associated pathways [100,101], important mechanisms in learning (mGluR and GABA), memory (mGluR) attention deficit and hyperactivity (dopamine).

Based on numerous studies on FXS aborted fetuses [50,102], FMRP is known to be still expressed, at least in part, in FXS fetuses during the first trimester, when most brain development takes place. Close to the start of the second trimester, *FMR1* epigenetic gene silencing occurs and FMRP is turned off. The absence of FMRP from that point on during development eventually leads to intellectual impairment, the clinical phenotype of FXS [103,104,105]. A long-standing unanswered question in the fragile X field relates to how the loss-of-function of FMRP causes FXS. In addition, there are questions as to precisely when FMRP expression becomes totally lost. In an attempt to address both of these issues, researchers have used brain samples obtained from aborted FXS fetuses [106,107]. These studies led to conflicting results, encouraging scientists to generate disease animal models to monitor the developmental regulated processes in FXS embryos. Both knockout and transgenic knockin mouse models for FXS have been created [15,16,18,20,22], but although *FMR1* knockout mice have been extremely informative for studying the clinical pathology of FMRP deficiency, they fail to recapitulate the course of the disease in a developmentally regulated context because they are FMRP protein deficient from day 1 [108]. On the other hand, when a human transgene of CGGs (PM range) was targeted into the 5′-UTR of *FMR1* by homologous recombination, the PM rarely expanded into the full mutation range in the offspring, and when it did, it often contracted and manifested almost no aberrant methylation, leaving the *FMR1* gene active [23]. These crucial issues underscore the need for complementary model systems that can reproducibly copy the underlying mechanisms of FXS. One approach would be to establish mutant PSCs, hESCs or patient-derived iPSCs, that are induced to differentiate into neural precursors (NPCs) and mature neurons in culture. In this respect, mutant PSCs may provide a great opportunity to track the timing and consequences of FMRP suppression by affording the exploration of disease relevant tissues and/or developmental stages that are otherwise inaccessible to research. Thus far, only a limited amount of work has been carried out on the neurological facets of the disease using mutant PSCs as a model system. The first attempt to use such cell types to generate post mitotic neurons and glia cells was by Sheridan et al. [38], who established iPSCs from a mosaic patient with PM and FM alleles. They created *FMR1* expressing (PM) and un-expressing (FM) isogenic iPSC clones, and compared their neural differentiation properties. Whereas FM iPSCs (methylated and inactive *FMR1*) exhibited fewer *Tuj1*-positive cells and shorter branch processes, in glial cells no clear difference could be detected between PM and affected clones. However, since *FMR1* is already inactive from the start in the FXS iPSCs, it may not properly reflect the molecular cascade that leads to the pathology as it occurs in vivo. On the other hand, using three different male FXS hESC lines as a substitute for patient-derived iPSCs, Telias et al. were able to generate electrophysiologically active cells using a differentiation protocol that enhanced the formation of fully developed neurons [44]. Although FXS neurons were able to create neuronal networks and showed no difference in voltage clamp recordings, significant differences in action potential properties such as spike rise time, duration, and spike number for a single depolarization event were detected. Importantly, these functional defects were detected only at the end stage of differentiation, and were coupled with the formation of fewer synaptic vesicles, a lack of spontaneous synaptic activity and reduced amplitudes of the action potentials [44,109]. By contrast, when FXS iPSCs were differentiated into forebrain neurons, researchers were able to show a clear reduction in the number and length of extensions as early as the neurite stage. Using time-lapse imaging of individual growth cones from control and FXS forebrain neurospheres, it was suggested that unlike in FXS hESCs, neurite initiation and extension are defective [35]. This may imply that the difference between FXS hESCs and iPSCs in the timing of epigenetic silencing of *FMR1* may be crucial for investigating the neural phenotype of the disease, since FXS iPSCs neurons are likely to present a more drastic phenotype than their FXS hESCs counterparts. On the other hand, as disease severity is fairly heterogeneous among patients, the expected phenotype may be highly variable between the different cell lines. This should be taken into account when trying to assess pathogenesis, and could be addressed by defining standard criteria for differentiation, and by verifying loss-of-function of the FMRP protein at equivalent differentiation stages.

Regarding the molecular mechanisms that are involved with impaired neurogenesis of FXS, it is hard to point to major downstream targets/specific pathways that play a central role in disease pathology since FMRP, as mentioned earlier, has many (hundreds to thousands [110]) downstream gene targets. This is why we still have almost no information on the key players that contribute to the major defects in FXS. In fact, only a handful of studies have been able to define potential gene candidates, and so far, none have been mechanistically confirmed. One such example is *RE1 Silencing Transcription Factor* (*REST*), which functions to repress neural genes in non-neuronal tissues. It is thought to be a master regulator in neurogenesis. By comparing gene expression in WT and affected FXS iPSCs, Halevy et al. provided a list the downregulated genes responsible for axon guidance that are typically controlled by *REST* [111]. Interestingly, *REST* itself is upregulated in FXS iPSC-derived neurons, most likely by the increase in *mir-382* mRNA levels. Rescue experiments by overexpression of *mir-382* in FXS iPSCs-derived neurons reduced elevated levels of *REST* and restored axon guidance related gene expression. A full characterization of the in vitro derived neurons in terms of subtype and degree of maturation were not reported. Another candidate is the SOX family of transcription factors, and in particular *SOX9* and *SOX2*, which are known for their key role in neurogenesis and are both highly expressed in NPCs [112,113]. By comparing expressions of mature in vitro differentiated neurons and FXS and WT hESCs, Telias et al. typically found a drop in FXS NPCs in *SOX9* expression levels concurrent with a rise in *SOX2* mRNA levels [114]. These results may suggest that FMRP regularly controls neural development by governing the delicate equilibrium between SOX2 and SOX9, and that in its absence, the imbalance between both of these transcription factors may lead to delayed or reduced neurogenesis. More research is required to establish whether FMRP controls *SOX2* and *SOX9* expression directly or indirectly, and to better understand the overall involvement of the SOX superfamily in the neuron defects associated with FXS. Finally, in a subsequent study the researchers used expression analysis for the GABA-A receptor subunits and reported increased levels of α2 subunit mRNA levels, which could possibly explain the lack of mature response to GABA signaling [115]. This latter finding is in line with previous results on the disruption of the GABA signaling pathway in mouse models and patients [100,116,117,118,119,120], hence confirming the authenticity of this model system. 

Neuronal differentiation protocols are improving and thus becoming more available to research labs worldwide. Apart from shortening the protocols considerably, it facilitates our ability to manipulate the cells to differentiate into specific lineages and particular subtypes of neurons in a more controlled and efficient fashion which will enable better evaluation. In addition, large scale gene expression profiles at various time points during nerve cell differentiation are expected to provide new insights and perhaps the identification of new key players in FXS pathogenesis. Hopefully, this will eventually lead to the development of new therapeutic approaches to cure, or more likely to relieve disease symptoms.

## 5. Therapeutic Strategies in FXS

Given the large number of mRNAs targeted by FMRP and the various known dysregulated pathways, including the GABAergic pathway (reviewed in [121]), clearly there would be advantages to being able to once again turn on the *FMR1* gene silenced by the FM. In fact, the existence of rare unmethylated full mutation individuals [122], suggests that pharmacologically restoring *FMR1* transcription may be possible. 

FXS is a neurodevelopmentally regulated condition which, like many other neurodevelopmental disorders, does not have a cure. To date, most efforts directed towards the development of drugs for FXS have focused on reducing the signs and symptoms of the disease rather than dealing with its cause (FMRP deficiency) [123]. However, since *FMR1* silencing is produced by epigenetic modifications and the coding sequence for *FMR1* remains intact in patients, scientists are aiming to develop alternative therapeutic approaches to treat patients by developing strategies to re-activate the gene and restore its function. Mutant PSCs provide a powerful platform for drug screening and development since they can potentially differentiate into all cell types in large numbers so that targeted tissues are available for large-scale drug screening (reviewed in [124]). This is particularly important in the case of FXS where the disease relevant cell types – neurons - are inaccessible for research apart from aborted fetuses or post mortem brain samples. FXS iPSCs-derived neurons are particularly informative since they retain epigenetic memory [39] and therefore are at all times completely methylated, reflecting the final state of gene inactivation in the patients’ somatic cells. In addition, hESCs is a convenient tool for genetic manipulation by targeted mutagenesis, since they provide a valuable instrument for developing gene therapy based approaches to FXS. 

Over the last few years there have been several attempts to use in vitro NPCs and fully matured in vitro differentiated neurons from FXS PSCs (mainly iPSCs) to pinpoint the chemical reagents that can efficiently remove the repressive epigenetic marks that are wrongly acquired by the mutation. At first, researchers aimed to explore the effect of a few Food and Drug Administration (FDA)- approved chromatin remodeling compounds including the general histone deacetylase inhibitor trichostatin-A (TSA) and the de-methylating agents 5-azacytidine (5-azaC) and its deoxy derivative, 5-aza-2′-deoxycytidine (5-aza-dC), which at low doses lead to the loss of DNA methylation by inhibition of DNA methyltransferases [125]. Taking advantage of FXS iPSCs derived from three different patients, they examined whether these compounds could re-activate the otherwise silenced *FMR1* gene in iPSCs and in vitro differentiated neurons [126]. The effect of the compounds was evaluated by the upregulation of *FMR1* mRNA levels, and was validated by immunostaining for FMRP. However, only 5-azaC was able to robustly reactivate gene expression in FXS neurons, although its association with TSA treatment led to a slight increase in mRNA levels. At high concentrations (10 µM) the effect of 5-azaC on DNA methylation reached 57% and was coupled with enrichments in H3K4me3 and H3-acetylation (active histone modification marks) in the neurons, and to a lesser extent in undifferentiated FX iPSCs. However, *FMR1* mRNA and protein levels were only partially restored (15%–45% mRNA levels of WT control). This may have resulted from the inability of 5-azaC to remove the repressive methylation mark of H3K9me3 from the locus, and is consistent with previously established data in patients’ somatic cells where *FMR1* re-activation only persisted for a limited time (5–7 weeks) after drug withdrawal. It is worth noting that following drug treatment massive cell death was observed in the undifferentiated FXS and WT iPSCs, most likely since the drug concentrations were beyond physiological levels found in the plasma of cancer patients that were treated with this agent [127]. An additional concern relates to the necessity for cell proliferation for the drug to be effective. This may be a crucial point when considering the use of this drug in post-mitotic neurons. Finally, it should be noted that 5-azaC has a general effect on methylation in the genome and therefore is likely to have an undesirable effect on global gene expression and the re-activation of dormant retro-elements distributed throughout the genome [128,129,130]. Overall, more safety and efficacy issues need to be addressed before this drug can be seriously considered for clinical application in FXS patients.

A more general and promising approach would be to screen for a large number of compounds in a way that does not rely on the best candidate approach and/or educated speculation. This could be done by high-throughput screenings to identify compounds that increase FMRP expression in FXS iPSCs-derived neurons. To date, three high-throughput screens based on iPSCs have been reported for *FMR1* re-activation. Using NPCs derived from the iPSCs of a single patient, Kumari and colleagues screened a collection of approximately 5000 known FDA approved drugs by a highly sensitive fluorescent detection assay for FMRP expression [37]. They identified 6 compounds that only slightly increased FMRP expression; however, none of these compounds resulted in clinically pertinent *FMR1* mRNA levels. Nevertheless, this study provided a proof-of-principle for how FXS iPSCs could be exploited as a platform for high-throughput screening to identify top compounds for FXS therapy. In a different screen, Kaufmann et al. tested a set of 50,000 compounds, including modulators of several epigenetic targets, with a high-content imaging system that measures FMRP, the most significant readout for the expected phenotype [36]. This enabled the identification of a number of compounds that led to FMRP expression, although the levels induced were far from those expressed in the WT control. Unexpectedly, the most promising compounds were those that induced FMRP expression in the cytoplasm, rather than chromatin remodeling factors. In a third study researchers created a clever reporter system that relies on knockin of a luciferase gene into the endogenous *FMR1* locus in FXS iPSCs [131]. By carrying high-throughput screen on NPCs derived from those reporter cell lines, it should be possible in the future to identify small molecules that will re-activate the *FMR1* gene. Overall, although identifying the mode of action of leading compounds were beyond the scope of these studies, they provided a proof-of-concept for how patient derived NPCs may pave the way for the discovery of new drugs and/or approaches to correct the FXS phenotype. That said, it is uncertain whether any molecule will ever be gene-specific and safe. Therefore, the ultimate therapy for FXS will be to specifically target the *FMR1* gene rather than treating patients with agents that act globally on the genome. 

A different approach to consider would be to target the locus by gene manipulation. Given that demethylation of *FMR1* by 5-azaC fails to remove the repressive mark H3K9me3, along with its inability to fully restore *FMR1* mRNA expression levels, it may be impossible to re-establish the transcriptional activity of the gene permanently as long as the extra CGGs remain. To explore whether re-activation can be achieved through the elimination of the repeats, Park et al. managed to delete the entire region by gene editing [73]. Using the CRISPR/Cas9 system with a single guided RNA targeted upstream to the repeats, they removed the CGGs from non-expressing and fully methylated FXS iPSCs clones with some of their flanking regions (20–60 bp upstream and 3–20 bp downstream to the repeats). Removal of the repeats led to a dramatic loss of methylation at the promoter, re-activation of the silenced gene and restored *FMR1* mRNA to normal levels. This was accompanied by a gain in H3K4me3 and a significant decrease in H3K9me3. Interestingly, when comparing gene expression profiles between the isogenic edited and non-edited FXS neurons, differential expression of glutamate receptor genes were found, which is consistent with the deregulation of glutamate signaling pathway in FXS [132]. Altogether, the researchers were able to show that genotypic correction by CGG deletion restored the function of the gene in FXS iPSCs and their neural cell derivatives. It remains to be determined whether complete elimination of the repeats, rather than shortening them to the normal range, has an adverse effect on the biological function of transcription factor binding as well as on the various RNA binding proteins it normally interacts with [133]. Moreover, off-target effects should be systematically evaluated throughout the genome, considering the ability of the guide RNA to target other sequences in the genome. Given the rapid advances in the field of gene editing and gene therapy, it may be possible in the near future to overcome this obstacle by recruiting particular chromatin remodeling factors (such as the catalytic domain of the demethylating enzymes TET1, 2 or 3) [134] specifically to the *FMR1* locus, and thus consistently remove the epigenetic modifications that are repeatedly deposited due to the expansion. 

## 6. Concluding Remarks

Despite the identification of the CGG expansion as the cause of FXS over two decades ago, little is known about the mechanisms underlying FXS. For example, it is still unclear how CGG expansion leads to epigenetic gene silencing of the *FMR1* gene, how the loss-of-function of FMRP leads to neural deficits in FXS fetuses, or how the CGGs become extensively unstable in patients. This is, in part, because currently available animal models do not accurately reproduce the molecular and cellular phenotypes of FXS in humans, and since the diseased organ—the developing brain—is rarely available for research. Hence, there is a need to complement human-based model systems in a way that can overcome the difficulties associated with obtaining a sufficient supply of disease relevant cells for basic and applied research. These systems should also bypass the need for gene targeting since lengthy repeat expansions are particularly difficult to artificially induce and maintain. For all of these reasons researchers are currently relying on mutant pluripotent stem cells, hESCs and iPSCs, as a way to address some of the gaps in our knowledge on the molecular mechanisms that go wrong in FXS developing fetuses. Thus far, over a dozen FXS hESC lines and many patient-derived iPSC clones have been established. These exclusive cell lines are expected to be particularly useful because they may provide an exceptional opportunity to look at affected neurons while they differentiate and become functionally mature. In addition, they provide a valuable way to genetically intervene and carry out functional assays and rescue experiments while they propagate and differentiate in vitro. Finally, they are expected to be a powerful tool in the development of new therapeutic approaches to correct, or more likely reduce, disease symptoms by searching for drug targets or by genetic engineering. However, we still need to better define how well these cell lines mimic the molecular and cellular pathways that are disturbed in vivo, and better evaluate the differences between FXS hESCs and patient-derived iPSCs, to be able to best exploit them to respond to the remaining unanswered questions regarding FXS.

## Figures and Tables

**Table 1:** Fragile X syndrome (FXS) Human embryonic stem cell lines.

Cell Line	Karyotype	Number of CGG Repeat	Methylation State	Derivation Center
SI-214	46, XY	138, 450	mostly methylated	RGI [40]
Lis01_HEFX1	46, XY	200–650	unmethylated	TASMC [27]
HAD5	46, XX	300	NA	HHUMC [41]
VUB11_FXS	46, XX	2000, 2010	fully methylated	VUB [42]
VUB13_FXS	46, XX	2000	fully methylated	VUB [42]
STR-189-FRAXA	46, XX	NA	NA	IGBMC [43]
STR-233-FRAXA	46, XY	NA	NA	IGBMC [43]
Lis26_FXS_6	46, XY	50–600	partially methyalted	TASMC [44]
WCMC37	46, XY	142, 167, 179, 450	mostly methylated	WCMC, [45]
SZ-FX1	46, XX	300–600	mostly methyalted	SZMC [32]
SZ-FX3	46, XX	300–600	partially methyalted	SZMC [32]
SZ-FX6	46, XY	200–600	partially methyalted	SZMC [32]
SZ-FX7	46, XX	200–300	unmethylated	SZMC [32]
SZ-FX8	46, XY	200–600	mostly methyalted	SZMC [32]
SZ-FX12	46, XX	150–300	partially methyalted	SZMC [32]
SZ-FX14	46, XY	290–600	mostly methyalted	SZMC [32]

Sixteen different embryonic stem cell lines were established from human embryos with a full mutation (>200 repeats) in the *fragile X mental retardation 1 (FMR1)* gene. The embryos were obtained from premutation female carriers through a preimplantation genetic diagnosis (PGD) procedure. The number of CGG repeats and the methylation status of the *FMR1* gene were determined at the time of derivation by various methodologies, and may have changed during in vitro culturing. NA, not available; RGI, Reproductive Genetics Institute; TASMC, Tel Aviv Sourasky Medical Center; HHUMC, Hadassah Hebrew University Medical Center; VUB, Vrije Universiteit Brussel; IGBMC, Institut Génétique Biologie Moléculaire Cellulaire; WCMC, Weill Cornell Medical College; SZMC, Shaare Zedek Medical Center.
